# Supplementary material for: Identifying anaphylaxis using weakly-supervised prediction models and natural language processing
Source: medRxiv. 2026 Jun 17:2026.06.09.26355005. Preprint. [Version 1] doi: 10.64898/2026.06.09.26355005 (PMC13308432; doi:10.64898/2026.06.09.26355005)
Supplement: Supplement 1 [file media-1.pdf]

LLT-CUI	LLT	PT-CUI	PT
C0000729	Griping abdomen	C0000729	Abdominal Pain
C0221512	Stomach ache		
C0232488	Abdominal colic		
C0232491	Chronic abdominal pain		
C0344304	Abdominal pain generalized		
C0423644	Central abdominal pain		
C0522061	Abdominal pain localized		
C0549273	Abdominal pain aggravated		
C0857092	Duodenal ulcer-type symptoms		
C0857334	Colicky		
C0920154	Spleen pain		
C1096624	Periumbilical pain		
C1609533	Functional abdominal pain		
C1662979	Peritoneal pain		
C1698089	Postprandial pain		
C3805159	Mesogastric pain		
C0340865	Anaphylactoid shock	C0000729	anaphylaxis
C0344159	Anaphylactic reaction to venom		
C0344183	Exercise-induced anaphylaxis		
C0685898	Anaphylactic shock due to adverse food		
C0857035	Acute anaphylaxis		
C1096621	Anaphylactic reaction to chemical		
C1536101	Anaphylactic reaction to vaccine		
C5441893	Anaphylactic reaction to drug		
C5442289	Anaphylactic reaction to contrast agent		
C5577808	Biphasic anaphylactic reaction		
C0549440	Angioneurotic oedema aggravated		
C0743747	Face angioedema		
C0857036	Acute angio oedema		
C0857323	Angioedema of larynx		
C0859055	Oedema vascular		

C0863128 Angioedema aggravated<br>C0877772 Angioneurotic edema, not elsewhere cla<br>C0920175 Allergic angioedema<br>C1304200 Lip angioedema<br>C4324658 Eye angioedema<br>C4324659 Respiratory angioedema	**C0002994**	**Angioedema**
C0154455 Other anxiety states<br>C0233481 Worry<br>C0233485 Apprehension<br>C0235107 Anxiety-ridden<br>C0235111 Anxiety complex<br>C0262377 Situational anxiety<br>C0349231 Phobia, unspecified<br>C0542180 Reaction alarm<br>C0549258 Angor animi<br>C0549259 Anxiety aggravated<br>C0581386 Chronic anxiety<br>C0683278 Anguish<br>C0700031 Anxiety attack<br>C0700613 Anxiety state<br>C0853074 Anxiety NEC<br>C0853241 Exacerbation of anxiety<br>C0857102 Feeling of intense apprehension<br>C0857126 Impending doom<br>C0857480 Made anxiety worse<br>C0859031 Face anguish<br>C0860603 Anxiety symptoms<br>C2239195 Anxious mood<br>C2945761 Alarm (not alarm reaction)	**C0003467**	**Anxiety**
C0155877 Atopic asthma<br>C0155878 Extrinsic asthma without mention of stat<br>C0155880 Intrinsic asthma		

C0155881	Intrinsic asthma without mention of stat		
C0155886	Asthma, unspecified type, without ment		
C0340067	Drug-induced asthma		
C0340076	Eosinophilic asthma		
C0347950	Asthmatic attack		
C0349790	Exacerbation of asthma		
C0549336	Asthma aggravated		
C0858626	Asthmatic attack induced		
C0859987	Asthmatoid bronchitis	**C0004096**	**Asthma**
C0877430	Asthma chronic		
C0877446	Asthma-like condition		
C0948683	Asthmatic attack atopic		
C1273489	Nocturnal asthma		
C1319018	Wheezy bronchitis		
C1735623	Cold induced asthma		
C2363774	Neutrophilic asthma		
C2363819	Paucigranulocytic asthma		
C2747883	Infection induced asthma		
C2919352	Seasonal asthma		
C5394677	Thunderstorm asthma		
C0015263	Exercise-induced bronchospasm		
C0079043	Bronchoconstriction	**C0006266**	**Bronchospasm**
C0235906	Bronchospasm aggravated		
C1609536	Allergic bronchospasm		
C0029537	Other chest pain		
C0151826	Substernal chest pain		
C0232286	Chest pain precordial		
C0232288	Chest pain exertional		
C0235710	Chest discomfort		
C0235715	Chest aching of		
C0235716	Chest burning pain of		
C0241243	Sternal pain		

C0302355	Chest pain NEC	C0008031	Chest Pain
C0522051	Acute chest pain		
C0541828	Chest pain-L arm		
C0740396	Chest burning		
C0856134	Chest pain aggravated		
C0856578	Parasternal pain (excluding organic)		
C0857030	Ache across chest		
C0857367	Generalized chest pain		
C1112757	Chest pain with radiation to left arm		
C1740831	Chronic chest pain		
C3203733	Precordial catch syndrome		
C0152124	Reactive confusion	C0009676	Confusion
C0154333	Subacute confusional state		
C0338642	Toxic confusional state		
C0813178	Bewilderment		
C0856071	Confusion aggravated		
C0856480	Confusional state (organic, acute or suba		
C0857042	Acute onset of confusion		
C0857386	Nocturnal confusion		
C0858851	Confusion reversible		
C1285577	Acute confusion state		
C0010201	Chronic cough	C0010200	Coughing
C0231911	Paroxysmal cough		
C0231912	Nocturnal cough		
C0264345	Smoker's cough		
C0423730	Painful cough		
C0455777	Cough ineffective		
C0562483	Persistent cough		
C0574067	Cough increased		
C0742857	Acute cough		
C0850149	Dry cough		
C0856112	Cough aggravated		

C0857172	Persistent dry cough		
C0857173	Persistent non-productive cough		
C0857513	Cough resembling asthma		
C0859870	Irritative cough		
C1096640	Coughing after drug inhalation		
C1142295	Drug-induced cough		
C2202983	Cough weak		
C4552485	Unexplained chronic cough		
C4552486	Refractory chronic cough		
C0156173	Functional diarrhea	**C0011991**	**Diarrhea**
C0239182	Watery diarrhoea		
C0267556	Osmotic diarrhea		
C0267557	Secretory diarrhea		
C0401151	Chronic diarrhea		
C0578159	Antibiotic-associated diarrhea		
C0740441	Acute diarrhea		
C0853947	Diarrhoea aggravated		
C0856217	Functional diarrhoea (due to spastic colo		
C0857098	Explosive diarrhoea		
C0857138	Frank diarrhoea		
C0857184	Urgent diarrhoea		
C0859971	Idiopathic diarrhoea		
C0859975	Nocturnal diarrhoea		
C0860247	Iatrogenic diarrhoea		
C0860527	Mushy stool		
C1868939	Mushy diarrhea		
C2129214	Loose bowels		
C2747921	Malodorous diarrhea		
C3671554	Mucous diarrhea		
C0039070	Syncope		
C0220870	Lightheadedness		
C0476206	Dizziness and giddiness		

C0476207	Giddiness	C0012833	Dizziness
C0549239	Dizziness aggravated		
C0581879	Felt faint		
C0595963	Wooziness		
C0852858	Vertigo (excluding dizziness)		
C0857087	Dizzy spells		
C0857226	Swaying feeling		
C0857314	Felt giddy		
C5442487	Idiopathic dizziness		
C0030824	Penicillin allergy	C0013182	Drug Allergy
C0038757	Sulfonamide allergy		
C0571622	Insulin allergy		
C0741103	Allergy to antibiotic		
C0850093	Allergic reaction to antibiotics		
C0857594	Specific allergy (drug)		
C3267191	Allergic reaction to analgesics		
C4708098	Allergy to vitamin B12		
C4727850	Allergy to topical drugs		
C5400282	Allergy to ayurvedic drugs		
C5400285	Allergy to allopathic drugs		
C5400286	Allergy to homeopathic drugs		
C5442512	Allergic reaction to chemotherapy		
C5553031	Allergy to NSAIDs		
C0029601	Other dyspnea and respiratory abnorma	C0013404	Dyspnea
C0159053	Dyspnea and respiratory abnormalities		
C0231848	Air hunger		
C0425449	Gasping		
C0553668	Labored breathing		
C0574066	Increased shortness of breath		
C0743323	Acute dyspnea		
C0853326	Dyspnoea exacerbated		
C0857142	Marked inactivity of chest wall on inspira		

C0859927	Increased work of breathing		
C0860550	Respiratory tract closed sensation of		
C0234940	Inflammatory oedema reaction	C0013604	Edema
C0238094	Idiopathic edema		
C0333241	Chronic edema		
C0333243	Pitting edema		
C0333244	Transient edema		
C0856110	Oedema aggravated		
C0857441	Oedematous weight gain		
C0858531	Oedema-like		
C2938876	Weeping edema		
C0014238	Parasitic endophthalmitis NOS	C0014236	Endophthalmitis
C0029610	Other endophthalmitis		
C0154773	Acute endophthalmitis		
C0154774	Chronic endophthalmitis		
C0259800	Purulent endophthalmitis		
C0859152	Other specified endophthalmitis		
C1532322	Infectious endophthalmitis		
C0159038	Pallor and flushing	C0016382	Flushing
C0856089	Flushing aggravated		
C0857115	Gross flushing		
C0857370	Generalized flushing		
C0860135	Flushed chest		
C0559469	Egg allergy		
C0559470	Peanut allergy		
C0577620	Allergy to nuts		
C0577625	Shellfish allergy		
C0685900	Seafood allergy		
C0685901	Fruit allergy		
C0856904	Fish allergy		
C0856905	Potato allergy		
C0857593	Ovalbumin allergy		

C0859949	Meat allergy	C0016470	Food Allergy
C1096643	Allergy to grains		
C1096735	Allergy to legumes		
C1112677	Protein allergy		
C1630645	Alpha-gal allergy		
C1690580	Allergic reaction to food		
C2363729	Spice allergy		
C2938930	Food allergen sensitisation		
C4075590	Soy allergy		
C4082933	Allergy to animal		
C4552514	Vegetable allergy		
C4761189	Allergy to edible fungus		
C5400281	Oil allergy		
C5577936	Sesame allergy		
C0857375	Hypoxic arrest	C0018790	Cardiac Arrest
C0858523	Cardiac arrest transient		
C0858981	Congestive cardioplegia		
C4285916	Ventricular standstill		
C0235699	Red neck syndrome	C0020517	Hypersensitivity
C0235893	Allergy aggravated		
C0282504	Environmental allergy		
C0375321	Upper respiratory tract hypersensitivity r		
C0413234	Acute allergic reaction		
C0869403	Allergy, unspecified, not elsewhere class		
C1527304	Allergic reaction		
C1736167	Systemic allergic reaction		
C0155800	Chronic hypotension		
C0375314	Iatrogenic hypotension		
C0476454	Blood pressure reading low		
C0520541	Hypotensive episode		
C0740481	Hypotension asymptomatic		
C0745176	Acute hypotension		

C0856090 Hypotension aggravated<br>C0857353 Hypotensive<br>C0858763 Hypotension paroxysm<br>C0858766 Blood pressure dropped transient<br>C0861177 Mean blood pressure decreased<br>C0863113 Hypotension symptomatic<br>C0948686 Preshock<br>C1699115 Transient systolic hypotension	**C0020649** **Hypotension**
C0021381 Exudative inflammation<br>C0234939 Inflammatory swelling<br>C0522570 Inflammation localized	**C0021368** **Inflammation**
C0021925 Intubation<br>C0857231 To facilitate intubation<br>C0860359 Reintubate<br>C1868878 Endotracheal reintubation	**C0021932** **Endotracheal intubation**
C0201682 Chemistry NOS	**C0022885** **Laboratory Procedures**
C0235552 Subglottic edema<br>C0264315 Glottic edema<br>C0392319 Vocal cord edema<br>C0472519 Reinke's edema<br>C1142210 Aryepiglottis oedema<br>C1735912 Acute laryngeal edema<br>C5554320 Pharyngolaryngeal edema	**C0023052** **Laryngeal Edema**
C1112473 Mast cell aggregation	**C0024899** **Mastocytosis**
C0375548 Nausea alone<br>C0549280 Nausea aggravated<br>C0746783 Postprandial nausea<br>C0857071 Churning of stomach<br>C0857218 Sickness/nausea<br>C1112312 Nausea post chemotherapy<br>C3495909 Nausea post radiotherapy	**C0027497** **Nausea**

C3854611	Anticipatory nausea		
C0085668	Secondary carcinoma	C0027627	**Neoplasm Metastasis**
C0153675	Secondary malignant neoplasm of respir		
C0153684	Secondary malignant neoplasm of other		
C0856617	Secondary carcinoma (known primary)		
C0859898	Secondary malignant neoplasm of other		
C5554011	Synchronous metastases		
C5554406	Metachronous metastasis		
C0030200	Intractable pain	C0030193	**Pain**
C0150055	Chronic pain		
C0184567	Acute pain		
C0234230	Pain burning		
C0234238	Ache		
C0234250	Referred pain		
C0234252	Mechanical pain		
C0234253	Pain at rest		
C0234254	Radiating pain		
C0235048	Smarting		
C0278144	Pain dull		
C0278146	Shooting pain		
C0278148	Throbbing pain		
C0281856	General body pain		
C0423603	Pricking pain		
C0520962	Pain localized		
C0520963	Diffuse pain		
C0542135	pain left side		
C0677500	Stinging		
C0853946	Pain exacerbated		
C0858802	Burn lesion pain transient aggravation of		
C0858803	Pain irritated		
C0858805	Prick pain feeling		
C0948843	Irradiating pain		

C0948844 Lancinating pain<br>C1317590 Trigger point pain<br>C2363726 Pain upon movement<br>C3805255 Residual pain<br>C4087510 Kinesalgia<br>C4523910 Pain post chemotherapy<br>C4524193 Pantalgia		
C0039231 Tachycardia<br>C0235240 Heart pounding<br>C0549267 Palpitations aggravated<br>C4727920 Exercise induced palpitation	**C0030252**	**Palpitations**
C0157724 Pruritus and related conditions<br>C0157725 Other specified pruritic conditions<br>C0239653 Itchy feet<br>C0240941 Itchy scalp<br>C0475858 Generalized pruritus<br>C0856060 Pruritus aggravated<br>C0857130 Itchy legs<br>C0858706 Extremities itchy sensation of<br>C0858708 Itch burning<br>C0858709 Pruritus of both hands<br>C0919793 Pruritus breast<br>C0948847 Hairy skin itching<br>C1142396 Localized itching<br>C3670839 Pruritus facial<br>C4523818 Itchy upper limbs<br>C5208084 Periorbital pruritus<br>C5243975 Localised pruritus<br>C5577868 Chronic pruritus of unknown origin	**C0033774**	**Pruritus**
C0007203 Cardiopulmonary resuscitation	**C0035273**	**Resuscitation procedure**
C0029737 Other shock without mention of trauma		

C0157450	Shock during or following labor and deliv	C0036974	Shock
C0157451	Shock during or following labor and deliv		
C0157452	Shock during or following labor and deliv		
C0157453	Shock during or following labor and deliv		
C0157454	Shock during or following labor and deliv		
C0157455	Shock during or following labor and deliv		
C0159051	Shock without mention of trauma		
C0221477	Vasomotor collapse		
C0344329	Collapse		
C0349412	Refractory shock		
C0495170	Shock following abortion and ectopic and		
C0853340	Peripheral shutdown		
C0856621	Shock (excluding traumatic and specific c		
C0856661	Clinically shocked		
C0857075	Collapsed semi conscious & shocked		
C0859211	Shock during or following labor and deliv		
C0859274	Postoperative shock, not elsewhere class		
C0749874	Upper respiratory symptom	**C0037090**	**Respiratory symptom**
C0021881	Intracutaneous test	C0037296	Hypersensitivity skin testing
C0030646	Skin patch test		
C0430561	Prick test		
C5577824	PEG skin test		
C5577825	Polyethylene glycol skin test		
C0677600	Stridor inspiratory	**C0038450**	**Stridor**
C0159045	Swelling, mass, or lump in head and neck	C0038999	Swelling
C0235434	Swelling non-inflammatory		
C0235435	Tissue puffing		
C0423601	Feeling swollen		
C0476228	Localized superficial swelling, mass, or lu		
C0476313	Groin swelling		
C0541781	Arms swollen red hot		

C0578454 Neck swelling<br>C0749356 Swelling femoral<br>C0853619 Local swelling<br>C0858956 Swelling of dorsum manus			
C0042420 Vasovagal syncope<br>C0234434 Syncope hypotensive<br>C0234435 Cough syncope<br>C0234437 Syncope postural<br>C0235242 Syncope exertional<br>C0340850 Neurally mediated syncope<br>C0340855 Syncope micturition<br>C0340856 Defecation syncope<br>C0749201 Orthostatic syncope<br>C0751535 Cardiac syncope<br>C0751536 Syncope convulsive<br>C0856092 Syncope aggravated<br>C0877090 Orthostatic collapse<br>C2721582 Swallow syncope<br>C3854672 Arrhythmic syncope<br>C4049594 Cardiovascular syncope	**C0039070**	**Syncope**
C0549268 Tachycardia aggravated<br>C0749249 Wide complex tachycardia<br>C0856629 Tachycardia (excluding paroxysmal)<br>C0858524 Tachycardia nervous<br>C0858871 Heart sound accelerated<br>C1328539 Reflex tachycardia	**C0039231**	**Tachycardia**
C0312422 Blacked out<br>C0853202 Loss of consciousness NEC<br>C4087400 Transient loss of consciousness	**C0041657**	**Unconscious State**
C0413402 Adverse reaction to antibiotics	**C0041755**	**Adverse reaction to drug**
C0029839 Other specified urticaria		

C0149526 Allergic urticaria<br>C0221232 Welts<br>C0234935 Acute urticaria<br>C0263347 Urticaria drug-induced<br>C0750016 Generalized urticaria<br>C0853333 Urticaria aggravated<br>C0853691 Histamine-like rash<br>C0854430 Urticaria subcutaneous<br>C0856779 Acute allergic urticaria<br>C0856780 Pharmacological urticaria<br>C0857390 Generalized urticarial rash<br>C0858686 Rash urticaria-like<br>C0858687 Urticarial symptom<br>C0859045 Erythema urticarial<br>C1261981 Urticaria localised<br>C1276118 Urticaria recurrent<br>C1536459 Nettle rash<br>C4728195 Atopic urticaria	**C0042109**	**Urticaria**
C0027498 Nausea and vomiting<br>C0151791 Nausea vomiting and diarrhea<br>C0152165 Persistent vomiting<br>C0232599 Bilious vomiting<br>C0235250 Hyperemesis<br>C0267172 Habit vomiting<br>C0728950 Vomiting alone<br>C0856095 Vomiting aggravated<br>C0859028 Vomiting reflex<br>C0948239 Vomiting of medication<br>C0948394 Vomiting post chemotherapy<br>C0949067 Tablet in vomitus<br>C1112670 Vomiting post radiotherapy<br>C1142579 Blennemesis	**C0042963**	**Vomiting**

C1168325	Postprandial emesis		
C0231874<br>C0231875<br>C0392681<br>C0857522<br>C0877638<br>C1168179<br>C1735643	Wheezing inspiratory<br>Wheezing expiratory<br>Asthmatic wheezing<br>Increased wheeziness<br>Wheezing aggravated<br>Sibilus<br>Chronic wheezing	**C0043144**	**Wheezing**
C0266815<br>C3889086<br>C4758639	Cow's milk allergy<br>Milk protein allergy<br>Cow's milk protein allergy	**C0079840**	**Milk Allergy**
C0814177<br>C0854626<br>C2363849<br>C4048276	Noninvasive procedure<br>Ulcer management<br>Non-surgical treatment<br>Invasive procedure	**C0087111**	**Therapeutic procedure**
C0238977<br>C0856911<br>C0857229<br>C0858703<br>C1112766<br>C4049344<br>C4049484<br>C4280766<br>C5208051	Cheek swelling<br>Swelling lips & face<br>Swelling of face & neck<br>Swollen of face feeling<br>Forehead swelling<br>Eyebrow swelling<br>Swelling of jaw angle<br>Jaw swelling<br>Temple swelling	**C0151602**	**Facial swelling**
C0856668	Face & tongue oedema	**C0151610**	**Edema of the tongue**
C0863180	Respiratory arrest (excluding neonatal)	**C0162297**	**Respiratory arrest**
C0033144<br>C0445202<br>C0679699<br>C0679700	Primary prevention<br>Prophylactic<br>Secondary prevention<br>Tertiary prevention	**C0199176**	**Prophylactic treatment**
C0554804	Assisted ventilation		

C1868981	Invasive mechanical ventilation	C0199470	Mechanical ventilation
C1868982	Noninvasive mechanical ventilation		
C4760774	Transnasal humidified rapid-insufflation		
C0220854	Hyperpnea	C0231835	Tachypnea
C0858639	Respiration stimulated		
C1504512	Medication aspiration	C0232070	Foreign body aspiration
C0232285	Chest pressure sensation of	C0235710	Chest discomfort
C0232292	Chest tightness		
C0235711	Chest distressed feeling of		
C0235712	Chest fullness of		
C0438716	Chest pressure		
C0742339	Chest heaviness		
C0856135	Chest tightness aggravated		
C0857399	Retrosternal burning		
C0857400	Retrosternal discomfort		
C0858787	Chest abnormal feeling of		
C0858788	Chest anxiety feeling of		
C0858789	Chest hot feeling of		
C0858790	Chest strangled feeling of		
C1142568	Chest pressure exertional		
C5400139	Chest wall discomfort		
C0858939	Pharynx closed sensation of	C0236071	Constriction in throat
C0236066	Lips swelling non-specific	C0240211	Lip swelling
C0155910	Pulmonary congestion and hypostasis	C0242073	Pulmonary congestion
C0582411	Pulmonary venous congestion		
C0858975	Pulmonary configuration increased		
C1262246	Pulmonary stasis		
C1737267	Acute pulmonary congestion		
C0700292	Hypoxemia	C0242184	Hypoxia
C3203358	Hypoventilation		
C5400503	Transient nocturnal oxygen desaturation		

C5554231 Refractory desaturation<br>C5554263 Happy hypoxia<br>C5554264 Silent hypoxia	C0242184	Hypoxia
C1142195 Delayed anaphylactoid reaction	C0340865	Anaphylactoid Reaction
C0859943 Hereditary allergy<br>C0859957 Spontaneous allergy<br>C1504524 Allergic shiner	C0392707	Atopy
C0236003 Mucosal swelling	C0521481	Mucous membrane edema
C0856623 Orofacial oedema<br>C0856910 Oedema lips & face<br>C1096623 Maxillofacial oedema<br>C4552534 Forehead edema	C0542571	Facial edema
C0549433 Surgical intervention<br>C1735594 Stereotactic surgery<br>C5577945 Stab incision	C0543467	Operative Surgical Procedures
C0750310 Intravascular depletion<br>C4523878 Extracellular fluid volume decreased	C0546884	Hypovolemia
C0001889 Akinetic mutism<br>C0085628 Stupor<br>C0151559 Central nervous system depression<br>C0234439 Semi-coma<br>C0235070 Unconscious partial<br>C0235071 Arousal difficult<br>C0424532 Semi-conscious<br>C0542207 Sensorium decreased<br>C0683369 Consciousness clouding<br>C0877450 Obnubilation<br>C0877609 Alertness decreased<br>C1142529 Precoma	C0549249	Depressed Level of Consciousness
C0151885 Reaction aggravation<br>C4761010 Adverse reaction to product	C0559546	Adverse reactions

C0235217 Skin vasodilatation<br>C0424829 Distended blood vessels<br>C0553725 Skin vasodilating<br>C0856757 Dilated veins<br>C1262231 Capillarectasia<br>C1328540 Peripheral vasodilatation<br>C1959622 Vein distended	**C0595862**	**Vasodilation disorder**
C0241381 Throat burning sensation of<br>C0542075 Local throat irritation<br>C0562057 Burning in throat<br>C0858938 Pharynx burning sensation of<br>C0858940 Pharynx irritated sensation of<br>C0858941 Pharynx itchy sensation of<br>C0863112 Pharyngo-oral irritation<br>C4087337 Raw throat	**C0700184**	**Throat irritation**
C0232071 Food aspiration<br>C0877412 Aspiration of gastrointestinal contents ir<br>C2585629 Aspiration into trachea<br>C3665956 Vomit aspiration<br>C4324315 Silent aspiration<br>C4761191 Aspiration into bronchus	**C0700198**	**Pulmonary aspiration**
C0159065 Other symptoms involving abdomen and<br>C0859796 Other specified symptoms involving abdo	**C0740651**	**Abdominal symptom**
C4324301 Vocal cord spasm	**C0859897**	**Vocal cord dysfunction**
C0375668 Late effect of adverse effect of drug, me<br>C0851307 Certain adverse effects not elsewhere cla<br>C0877761 Other specified adverse effect, not elsew	**C0877248**	**Adverse event**
C0241955 Jellyfish sting	**C1096052**	**Venomous sting**
C0034076 Pulmonary insufficiency following traum<br>C0035229 Respiratory insufficiency<br>C0340194 Respiratory failure type 1		

C0398353	Hypercapnic respiratory failure	C1145670	Respiratory Failure
C0852895	Respiratory failures (excluding neonatal)		
C0877746	Other pulmonary insufficiency, not elsew		
C0948755	Pulmonary failure		
C2363912	Respiratory failure aggravated		
C3277226	Restrictive respiratory insufficiency		
C3805211	Hypoxic respiratory failure		
C4324356	End stage lung disease		
C0542098	Nasal mucus increased	C1260880	Rhinorrhea
C0558361	Sniffles		
C0858633	Nasal discharge watery excessive		
C1262304	Gustatory rhinorrhoea		
C0425481	Respiratory sighs	C1260922	Abnormal breathing
C0152229	Unattended death	C1306577	Death (finding)
C0277589	Death unexplained		
C0277608	Hospital death		
C0421619	Dead on arrival		
C0476463	Found dead		
C0857796	Died in sleep		
C1869004	Death from natural causes		
C3889051	Death unascertained		
C5400128	Death by homicide		
C0019825	Hoarse voice	C1527344	Dysphonia
C0029873	Other voice disturbance		
C0264614	Hypernasality		
C0264618	Hyponasality		
C0521007	Hypophonia		
C0751513	Rhinolalia		
C0854299	Disturbance in loudness		
C0857508	Distorted voice		
C0858567	Phonation difficulty		

C0858568 Voice lowered<br>C0860616 Resonance disorder<br>C0860618 Vocal tone disorder<br>C0860619 Vocal volume disorder<br>C1527340 Voice alteration		
C0021489 Intradermal injection<br>C0021490 Intralesional injection<br>C2957535 Intramuscular injection	**C1533685**	**Injection procedure**
C0856716 Asthma aspirin-sensitive<br>C1096641 Widal syndrome<br>C1328364 Analgesic asthma syndrome	**C3853540**	**Aspirin exacerbated respiratory disease**
C0375703 Anaphylactic shock due to unspecified fo<br>C0375707 Anaphylactic shock due to tree nuts and<br>C0391985 Anaphylactic shock, not elsewhere classi<br>C0858807 Drug shock<br>C0858808 Penicillin shock<br>C0859855 Anaphylactic shock due to peanuts<br>C0859856 Anaphylactic shock due to crustaceans<br>C0859857 Anaphylactic shock due to fruits and veg<br>C0859858 Anaphylactic shock due to fish<br>C0859859 Anaphylactic shock due to food additives<br>C0859860 Anaphylactic shock due to milk products<br>C0859861 Anaphylactic shock due to eggs<br>C3161345 Anaphylactic shock due to other specifie	**C4316895**	**Anaphylactic shock**
