# Supplementary material for: Identifying anaphylaxis using weakly-supervised prediction models and natural language processing
Source: medRxiv. 2026 Jun 17:2026.06.09.26355005. Preprint. [Version 1] doi: 10.64898/2026.06.09.26355005 (PMC13308432; doi:10.64898/2026.06.09.26355005)
Supplement: Supplement 2 [file media-2.pdf]

Supplementary Material to "Identifying anaphylaxis using weakly-supervised prediction models and natural language processing"

Brian D Williamson[1,2,3,*], David J Cronkite[1], Onchee Yu[1], Arvind Ramaprasan[1], Sharon Fuller[1], Jennifer Covey[1], Erika Kiniry[1], Daniel Park[4], Robert Winter[4], Jill Whitaker[4], Michael F. McLemore[4], Saranrat Wittayanukorn[5], Danijela Stojanovic[5], Yueqin Zhao[5], Sarah Dutcher[5], David S Carrell[1], Lisa A Jackson[1], Jennifer C Nelson[1,2], Joshua C Smith[4]

[1]Kaiser Permanente Washington Health Research Institute, Seattle, WA

[2]University of Washington, Seattle, WA

[3]Fred Hutchinson Cancer Center, Seattle, WA

[4]Vanderbilt University Medical Center, Nashville, TN

[5]US Food and Drug Administration, Silver Spring, MD

*corresponding author: brian.d.williamson@kp.org

S1: Supplemental Methods

S1.1: Anaphylaxis encounter path eligibility criteria

Table S1: ICD-10 diagnosis codes for anaphylaxis.

Code	Code description
T78.2XXA	Anaphylactic shock, unspecified, initial encounter
T88.6XXA	Anaphylactic reaction due to adverse effect of correct drug or medicament properly administered, initial encounter
T78.00XA	Anaphylactic reaction due to unspecified food
T78.01XA	Anaphylactic reaction due to peanuts
T78.02XA	Anaphylactic reaction due to shellfish (crustaceans)
T78.03XA	Anaphylactic reaction due to other fish
T78.04XA	Anaphylactic reaction due to fruits and vegetables
T78.05XA	Anaphylactic reaction due to tree nuts and seeds
T78.06XA	Anaphylactic reaction due to food additives
T78.07XA	Anaphylactic reaction due to milk and dairy products

T78.08XA	Anaphylactic reaction due to eggs
T78.09XA	Anaphylactic reaction due to other food products
T80.51XA	Anaphylactic reaction due to administration of blood/products, initial encounter
T80.52XA	Anaphylactic reaction due to vaccination, initial encounter
T80.59XA	Anaphylactic reaction due to other serum, initial encounter
T80.59XA	Anaphylactic reaction due to other serum, initial encounter

Table S2: Diagnosis and procedure codes for symptoms, procedures, or treatments that may occur on the same calendar day as an anaphylaxis diagnosis.

Group	Code	Code type	Code type and description
I	J9801	ICD-10	Bronchiospasm
	R061	ICD-10	Stridor
	J1200	HCPCS	Injection of diphenhydramine
II	I959	ICD-10	Hypotension
	J0170	HCPCS	Injection of epinephrine
	J0171	HCPCS	Injection of epinephrine
	92950	CPT	CPR procedure
	5A12012	ICD-10	CPR procedure
	5A1221Z	ICD-10	CPT procedure

Table S3: ICD-10 diagnosis codes for allergy unspecified, other unspecified adverse effects of drugs, medicinal and biological substances in therapeutic use.

Code	Code description
T50905A	Adverse effect of unspecified drugs, medicaments and biological substances, initial encounter
T410X5A	Adverse effect of inhaled anesthetics, initial encounter
T411X5A	Adverse effect of intravenous anesthetics, initial encounter
T41205A	Adverse effect of unspecified general anesthetics, initial encounter

T41295A	Adverse effect of other general anesthetics, initial encounter
T413X5A	Adverse effect of local anesthetics, initial encounter
T4145XA	Adverse effect of unspecified anesthetic, initial encounter
T8859XA	Other complications of anesthesia, initial encounter
T383X5A	Adverse effect of insulin and oral hypoglycemic [antidiabetic] drugs, initial encounter
T50995A	Adverse effect of other drugs, medicaments and biological substances, initial encounter
See Table S4	The set of 164 distinct ICD-10 codes that map to ICD-9 code 995.29 (“Unspecified adverse effect of other drug, medicinal or biological substance”).
T78.40XA	Allergy, unspecified, initial encounter
T78.49XA	Other allergy, initial encounter

Table S4: The set of 164 distinct ICD-10 codes that map to ICD-9 code 995.29 (“Unspecified adverse effect of other drug, medicinal or biological substance”).

Code	Description
T360X5A	Adverse effect of penicillins, initial encounter
T361X5A	Adverse effect of cephalosporins and other beta-lactam antibiotics, initial encounter
T362X5A	Adverse effect of chloramphenicol group, initial encounter
T363X5A	Adverse effect of macrolides, initial encounter
T364X5A	Adverse effect of tetracyclines, initial encounter
T365X5A	Adverse effect of aminoglycosides, initial encounter
T366X5A	Adverse effect of rifampicins, initial encounter
T367X5A	Adverse effect of antifungal antibiotics, systemically used, initial encounter
T368X5A	Adverse effect of other systemic antibiotics, initial encounter
T3695XA	Adverse effect of unspecified systemic antibiotic, initial encounter
T370X5A	Adverse effect of sulfonamides, initial encounter
T371X5A	Adverse effect of antimycobacterial drugs, initial encounter
T372X5A	Adverse effect of antimalarials and drugs acting on other blood protozoa, initial encounter
T373X5A	Adverse effect of other antiprotozoal drugs, initial encounter
T374X5A	Adverse effect of anthelminthics, initial encounter
T375X5A	Adverse effect of antiviral drugs, initial encounter
T378X5A	Adverse effect of other specified systemic anti-infectives and antiparasitics, initial encounter
T3795XA	Adverse effect of unspecified systemic anti-infective and antiparasitic, initial encounter
T380X5A	Adverse effect of glucocorticoids and synthetic analogues, initial encounter
T381X5A	Adverse effect of thyroid hormones and substitutes, initial encounter
T382X5A	Adverse effect of antithyroid drugs, initial encounter
T384X5A	Adverse effect of oral contraceptives, initial encounter
T385X5A	Adverse effect of other estrogens and progestogens, initial encounter
T386X5A	Adverse effect of antigonadotrophins, antiestrogens, antiandrogens, NEC, initial encounter
T387X5A	Adverse effect of androgens and anabolic congeners, initial encounter
T38805A	Adverse effect of unspecified hormones and synthetic substitutes, initial encounter
T38815A	Adverse effect of anterior pituitary [adenohypophyseal] hormones, initial encounter
T38895A	Adverse effect of other hormones and synthetic substitutes, initial encounter
T38905A	Adverse effect of unspecified hormone antagonists, initial encounter
T38995A	Adverse effect of other hormone antagonists, initial encounter
T39015A	Adverse effect of aspirin, initial encounter
T39095A	Adverse effect of salicylates, initial encounter

T391X5A	Adverse effect of 4-Aminophenol derivatives, initial encounter
T392X5A	Adverse effect of pyrazolone derivatives, initial encounter
T39315A	Adverse effect of propionic acid derivatives, initial encounter
T39395A	Adverse effect of other nonsteroidal anti-inflammatory drugs [NSAID], initial encounter
T394X5A	Adverse effect of antirheumatics, not elsewhere classified, initial encounter
T398X5A	Adverse effect of other nonopioid analgesics and antipyretics, NEC, initial encounter
T3995XA	Adverse effect of unspecified nonopioid analgesic, antipyretic and antirheumatic, initial encounter
T400X5A	Adverse effect of opium, initial encounter
T402X5A	Adverse effect of other opioids, initial encounter
T403X5A	Adverse effect of methadone, initial encounter
T404X5A	Adverse effect of other synthetic narcotics, initial encounter
T405X5A	Adverse effect of cocaine, initial encounter
T40605A	Adverse effect of unspecified narcotics, initial encounter
T40695A	Adverse effect of other narcotics, initial encounter
T407X5A	Adverse effect of cannabis (derivatives), initial encounter
T40905A	Adverse effect of unspecified psychodysleptics [hallucinogens], initial encounter
T40995A	Adverse effect of other psychodysleptics [hallucinogens], initial encounter
T415X5A	Adverse effect of therapeutic gasses, initial encounter
T420X5A	Adverse effect of hydantoin derivatives, initial encounter
T421X5A	Adverse effect of iminostilbenes, initial encounter
T422X5A	Adverse effect of succinimides and oxazolidinediones, initial encounter
T423X5A	Adverse effect of barbiturates, initial encounter
T424X5A	Adverse effect of benzodiazepines, initial encounter
T425X5A	Adverse effect of mixed antiepileptics, initial encounter
T426X5A	Adverse effect of other antiepileptic and sedative-hypnotic drugs, initial encounter
T4275XA	Adverse effect of unspecified antiepileptic and sedative-hypnotic drugs, initial encounter
T428X5A	Adverse effect of antiparkinsonism drugs & other central muscle-tone depressants, initial encounter
T43015A	Adverse effect of tricyclic antidepressants, initial encounter
T43025A	Adverse effect of tetracyclic antidepressants, initial encounter
T431X5A	Adverse effect of monoamine-oxidase-inhibitor antidepressants, initial encounter
T43205A	Adverse effect of unspecified antidepressants, initial encounter
T43215A	Adverse effect of selective serotonin and norepinephrine reuptake inhibitors, initial encounter
T43225A	Adverse effect of selective serotonin reuptake inhibitors, initial encounter
T43295A	Adverse effect of other antidepressants, initial encounter
T433X5A	Adverse effect of phenothiazine antipsychotics and neuroleptics, initial encounter
T434X5A	Adverse effect of butyrophenone and thiothixene neuroleptics, initial encounter
T43505A	Adverse effect of unspecified antipsychotics and neuroleptics, initial encounter
T43595A	Adverse effect of other antipsychotics and neuroleptics, initial encounter
T43605A	Adverse effect of unspecified psychostimulants, initial encounter
T43615A	Adverse effect of caffeine, initial encounter
T43625A	Adverse effect of amphetamines, initial encounter
T43635A	Adverse effect of methylphenidate, initial encounter
T43695A	Adverse effect of other psychostimulants, initial encounter
T438X5A	Adverse effect of other psychotropic drugs, initial encounter
T4395XA	Adverse effect of unspecified psychotropic drug, initial encounter
T440X5A	Adverse effect of anticholinesterase agents, initial encounter
T441X5A	Adverse effect of other parasympathomimetics [cholinergics], initial encounter
T442X5A	Adverse effect of ganglionic blocking drugs, initial encounter
T442X5S	Adverse effect of ganglionic blocking drugs, sequela
T443X5A	Adverse effect of other parasympatholytics and spasmolytics, initial encounter
T444X5A	Adverse effect of predominantly alpha-adrenoreceptor agonists, initial encounter
T445X5A	Adverse effect of predominantly beta-adrenoreceptor agonists, initial encounter
T446X5A	Adverse effect of alpha-adrenoreceptor antagonists, initial encounter
T447X5A	Adverse effect of beta-adrenoreceptor antagonists, initial encounter
T448X5A	Adverse effect of centrally-acting and adrenergic-neuron-blocking agents, initial encounter
T44905A	Adverse effect of unspecified drugs primarily affecting the autonomic nervous system, init. enc.
T44905S	Adverse effect of unspecified drugs primarily affecting the autonomic nervous system, sequela
T44995A	Adverse effect of other drug primarily affecting the autonomic nervous system, initial encounter
T44995S	Adverse effect of other drug primarily affecting the autonomic nervous system, sequela
T450X5A	Adverse effect of antiallergic and antiemetic drugs, initial encounter
T451X5A	Adverse effect of antineoplastic and immunosuppressive drugs, initial encounter

T452X5A	Adverse effect of vitamins, initial encounter
T453X5A	Adverse effect of enzymes, initial encounter
T454X5A	Adverse effect of iron and its compounds, initial encounter
T45515A	Adverse effect of anticoagulants, initial encounter
T45525A	Adverse effect of antithrombotic drugs, initial encounter
T45605A	Adverse effect of unspecified fibrinolysis-affecting drugs, initial encounter
T45615A	Adverse effect of thrombolytic drugs, initial encounter
T45625A	Adverse effect of hemostatic drug, initial encounter
T45695A	Adverse effect of other fibrinolysis-affecting drugs, initial encounter
T457X5A	Adverse effect of anticoagulant antagonists, vitamin K and other coagulants, initial encounter
T458X5A	Adverse effect of other primarily systemic and hematological agents, initial encounter
T4595XA	Adverse effect of unspecified primarily systemic and hematological agent, initial encounter
T460X5A	Adverse effect of cardiac-stimulant glycosides and drugs of similar action, initial encounter
T461X5A	Adverse effect of calcium-channel blockers, initial encounter
T462X5A	Adverse effect of other antidysrhythmic drugs, initial encounter
T463X5A	Adverse effect of coronary vasodilators, initial encounter
T464X5A	Adverse effect of angiotensin-converting-enzyme inhibitors, initial encounter
T465X5A	Adverse effect of other antihypertensive drugs, initial encounter
T466X5A	Adverse effect of antihyperlipidemic and antiarteriosclerotic drugs, initial encounter
T467X5A	Adverse effect of peripheral vasodilators, initial encounter
T468X5A	Adverse effect of antivaricose drugs, including sclerosing agents, initial encounter
T46905A	Adverse effect of unspecified agents primarily affecting the cardiovascular system, initial encounter
T46995A	Adverse effect of other agents primarily affecting the cardiovascular system, initial encounter
T470X5A	Adverse effect of histamine H2-receptor blockers, initial encounter
T471X5A	Adverse effect of other antacids and anti-gastric-secretion drugs, initial encounter
T472X5A	Adverse effect of stimulant laxatives, initial encounter
T473X5A	Adverse effect of saline and osmotic laxatives, initial encounter
T474X5A	Adverse effect of other laxatives, initial encounter
T475X5A	Adverse effect of digestants, initial encounter
T476X5A	Adverse effect of antidiarrheal drugs, initial encounter
T477X5A	Adverse effect of emetics, initial encounter
T478X5A	Adverse effect of other agents primarily affecting gastrointestinal system, initial encounter
T4795XA	Adverse effect of unspecified agents primarily affecting the gastrointestinal system, initial encounter
T480X5A	Adverse effect of oxytocic drugs, initial encounter
T481X5A	Adverse effect of skeletal muscle relaxants [neuromuscular blocking agents], initial encounter
T48205A	Adverse effect of unspecified drugs acting on muscles, initial encounter
T48295A	Adverse effect of other drugs acting on muscles, initial encounter
T483X5A	Adverse effect of antitussives, initial encounter
T484X5A	Adverse effect of expectorants, initial encounter
T485X5A	Adverse effect of other anti-common-cold drugs, initial encounter
T486X5A	Adverse effect of antiasthmatics, initial encounter
T48905A	Adverse effect of unspecified agents primarily acting on the respiratory system, initial encounter
T48995A	Adverse effect of other agents primarily acting on the respiratory system, initial encounter
T490X5A	Adverse effect of local antifungal, anti-infective and anti-inflammatory drugs, initial encounter
T491X5A	Adverse effect of antipruritics, initial encounter
T492X5A	Adverse effect of local astringents and local detergents, initial encounter
T493X5A	Adverse effect of emollients, demulcents and protectants, initial encounter
T494X5A	Adverse effect of keratolytics, keratoplastics, & other hair treatment drugs & prep's, initial encounter
T495X5A	Adverse effect of ophthalmological drugs and preparations, initial encounter
T496X5A	Adverse effect of otorhinolaryngological drugs and preparations, initial encounter
T497X5A	Adverse effect of dental drugs, topically applied, initial encounter
T498X5A	Adverse effect of other topical agents, initial encounter
T4995XA	Adverse effect of unspecified topical agent, initial encounter
T500X5A	Adverse effect of mineralocorticoids and their antagonists, initial encounter
T501X5A	Adverse effect of loop [high-ceiling] diuretics, initial encounter
T502X5A	Adverse effect of carbonic-anhydrase inhibitors, benzothiadiazides & other diuretics, initial encounter
T503X5A	Adverse effect of electrolytic, caloric and water-balance agents, initial encounter
T504X5A	Adverse effect of drugs affecting uric acid metabolism, initial encounter
T505X5A	Adverse effect of appetite depressants, initial encounter
T506X5A	Adverse effect of antidotes and chelating agents, initial encounter
T507X5A	Adverse effect of analeptics and opioid receptor antagonists, initial encounter

T508X5A	Adverse effect of diagnostic agents, initial encounter
T50905A	Adverse effect of unspecified drugs, medicaments and biological substances, initial encounter
T50A15A	Adverse effect of pertussis vaccine, incl. combinations with a pertussis component, initial encounter
T50A25A	Adverse effect of mixed bacterial vaccines without a pertussis component, initial encounter
T50A95A	Adverse effect of other bacterial vaccines, initial encounter
T50B15A	Adverse effect of smallpox vaccines, initial encounter
T50B95A	Adverse effect of other viral vaccines, initial encounter
T50Z15A	Adverse effect of immunoglobulin, initial encounter
T50Z95A	Adverse effect of other vaccines and biological substances, initial encounter
T887XXA	Unspecified adverse effect of drug or medicament, initial encounter

S1.3: Medical record abstraction form

See the supplementary data (“anaphylaxis_review_form_template_VUMC.xlsx”) accompanying this manuscript.

S1.4: Sampling strata

Table S5: Final sampling strata and sampling weights used at VUMC and KPWA.

Site	Path	Identifier	Vaccine-proximal	N eligible	N sampled	Sampling Weight
VUMC	1	---	---	904	165	5.479
	2	---	---	177	44	4.022
	3	---	---	202	45	4.489
KPWA	1	Even	0	237	27	8.778
	1	Even	1	9	9	1
	1	Odd	0	209	17	12.294
	1	Odd	1	12	12	1
	2	Even	0	189	30	6.3
	2	Even	1	6	6	1
	2	Odd	0	205	17	12.059
	2	Odd	1	5	5	1
	3	Even	0	67	9	7.444
	3	Even	1	3	3	1
	3	Odd	0	81	5	16.2
	3	Odd	1	5	5	1

S2: Supplemental Results

S2.1: UMLS CUIs discovered using AFEP

Table S6: Anaphylaxis-relevant Unified Medical Language System (UMLS) Concept Unique Identifers (CUIs) discovered using the Automated Feature Extraction for Phenotyping (AFEP) method.

CUI	Concept Name
C0000729	Abdominal cramps
C0000737	Abdominal pain
C0001883	Airways obstruction
C0002792	Anaphylaxis
C0002994	Angioedema
C0003467	Anxiety
C0004096	Asthma
C0005658	Bite wound
C0006266	Bronchospasm
C0007203	Cardiopulmonary resuscitation
C0008031	Chest pain
C0009443	Common cold
C0009676	Confusion
C0010200	Cough
C0011991	Diarrhea
C0012833	Dizziness
C0013182	Drug allergy
C0013404	Dyspnea
C0013604	Edema
C0014236	Endophthalmitis
C0014563	Epinephrine
C0015376	Extravasation
C0015663	Fasting
C0016382	Flushing
C0016462	Food contamination
C0016470	Food allergy
C0018790	Cardiac arrest
C0019825	Hoarseness
C0020517	Hypersensitivity
C0020523	Immediate hypersensitivity
C0020649	Hypotension
C0020683	Hypovolemic shock
C0021368	Inflammation
C0021564	Insect bite NOS
C0021925	Intubation
C0021932	Endotracheal intubation
C0022885	Laboratory test
C0023052	Laryngeal edema

C0024899	Mastocytosis
C0026821	Muscle cramp
C0027497	Nausea
C0027498	Nausea and vomiting
C0027627	Metastasis
C0028778	Obstruction
C0030193	Pain
C0030252	Palpitations
C0033774	Pruritus
C0035273	Resuscitation
C0036974	Shock
C0036980	Cardiogenic shock
C0037090	Respiratory symptom
C0037296	Skin test
C0038340	Sting
C0038450	Stridor
C0038999	Swelling
C0039070	Syncope
C0039231	Tachycardia
C0040533	Toxic effect of venom
C0041657	Loss of consciousness
C0041755	Adverse drug reaction
C0042109	Urticaria
C0042196	Vaccination
C0042420	Syncope vasovagal
C0042963	Vomiting
C0043144	Wheezing
C0079603	Immunofluorescence
C0079840	Milk allergy
C0087111	Therapeutic procedure
C0149783	Steroid therapy
C0151602	Facial swelling
C0151610	Tongue edema
C0155877	Allergic asthma
C0162297	Respiratory arrest
C0199176	Prophylaxis
C0199470	Mechanical ventilation
C0199747	Allergy test
C0202202	Protein

C0220787	Endotracheal aspiration
C0220870	Lightheadedness
C0221232	Welts
C0231835	Tachypnea
C0231848	Air hunger
C0232070	Foreign body aspiration
C0232292	Chest tightness
C0235710	Chest discomfort
C0236068	Swelling of tongue
C0236071	Throat constriction
C0238614	Exposure to allergen
C0240211	Lip swelling
C0242073	Pulmonary congestion
C0242184	Hypoxia
C0340865	Anaphylactoid reaction
C0344183	Exercise-induced anaphylaxis
C0347950	Asthmatic attack
C0349790	Exacerbation of asthma
C0392707	Atopy
C0413119	Wasp sting
C0413120	Bee sting
C0413234	Acute allergic reaction
C0426576	Gastrointestinal symptom NOS
C0442856	Hypoperfusion
C0476207	Giddiness
C0476273	Respiratory distress
C0521481	Mucosal edema
C0542571	Face edema
C0543467	Surgical procedure
C0546884	Hypovolemia
C0549249	Depressed level of consciousness
C0554804	Assisted ventilation
C0559469	Egg allergy
C0559470	Peanut allergy
C0559546	Adverse reaction
C0577620	Allergy to nuts
C0577628	Latex allergy
C0586407	Cutaneous symptom
C0595862	Vasodilatation

C0600228	Cardiopulmonary arrest
C0677500	Stinging
C0685898	Anaphylactic reaction to food
C0700184	Throat irritation
C0700198	Pulmonary aspiration
C0740651	Abdominal symptom
C0740852	Upper airway obstruction
C0743747	Face angioedema
C0744425	Glucocorticoid therapy
C0751535	Cardiac syncope
C0850569	Allergic rash
C0854051	Allergy to sting
C0854649	Anaphylaxis treatment
C0856904	Fish allergy
C0857035	Acute anaphylaxis
C0857353	Hypotensive
C0859897	Vocal cord dysfunction
C0877248	Adverse event
C0947961	Atopic disorders
C1096052	Venomous sting
C1145670	Respiratory failure
C1260880	Rhinorrhea
C1260922	Abnormal breathing
C1261392	Insect bite allergy
C1275515	Venomous bite
C1304200	Lip angioedema
C1306577	Death
C1328414	Blood tryptase
C1504322	Tryptase increased
C1504374	Antihistamine therapy
C1527304	Allergic reaction
C1527344	Dysphonia
C1533685	Injection
C1861783	Median arcuate ligament syndrome
C2939065	Airway edema
C3853540	Aspirin-exacerbated respiratory disease
C4047193	Epinephrine Auto-Injector
C4055482	Airway compromise
C4316895	Anaphylactic shock

C4324659	Respiratory angioedema
C4510560	Allergy to insect sting
C4728126	Gastrointestinal spasm
C5208132	Respiratory compromise

S2.2: Mapping between MedDRA preferred terms and associated child terms

See the supplementary data ("AFEP_normalization_mapping.xlsx") accompanying this manuscript.

S2.3: Supplemental figures

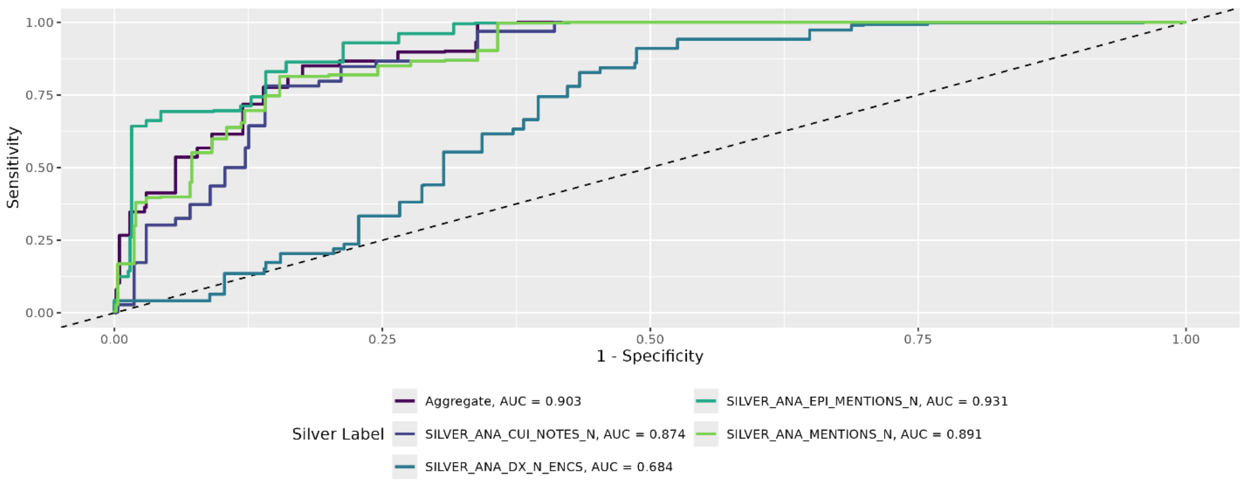

Figure S1: Receiver operating characteristic curves and AUCs for all models trained at KPWA and evaluated on data from KPWA. The silver labels are counts of anaphylaxis diagnosis codes (ANA_DX_N_ENCS), anaphylaxis mentions (ANA_MENTIONS_N), anaphylaxis concept unique identifiers (ANA_CUI_NOTES_N), and anaphylaxis or epinephrine mentions (ANA_EPI_MENTIONS_N).

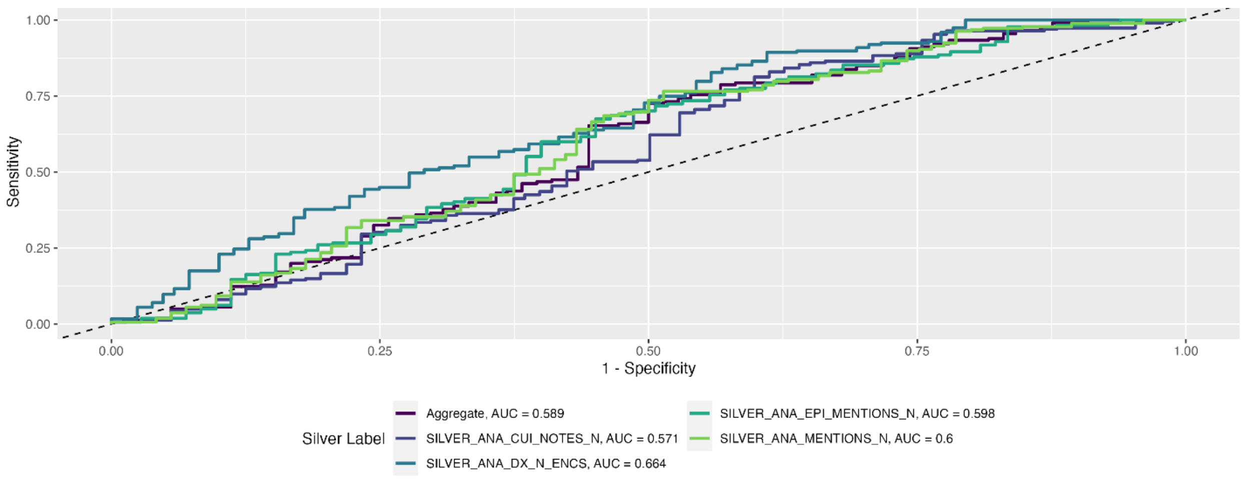

Figure S2: Receiver operating characteristic curves and AUCs for all models trained at VUMC and evaluated on data from VUMC. The silver labels are counts of anaphylaxis diagnosis codes (ANA_DX_N_ENCS), anaphylaxis mentions (ANA_MENTIONS_N), anaphylaxis concept unique identifiers (ANA_CUI_NOTES_N), and anaphylaxis or epinephrine mentions (ANA_EPI_MENTIONS_N).

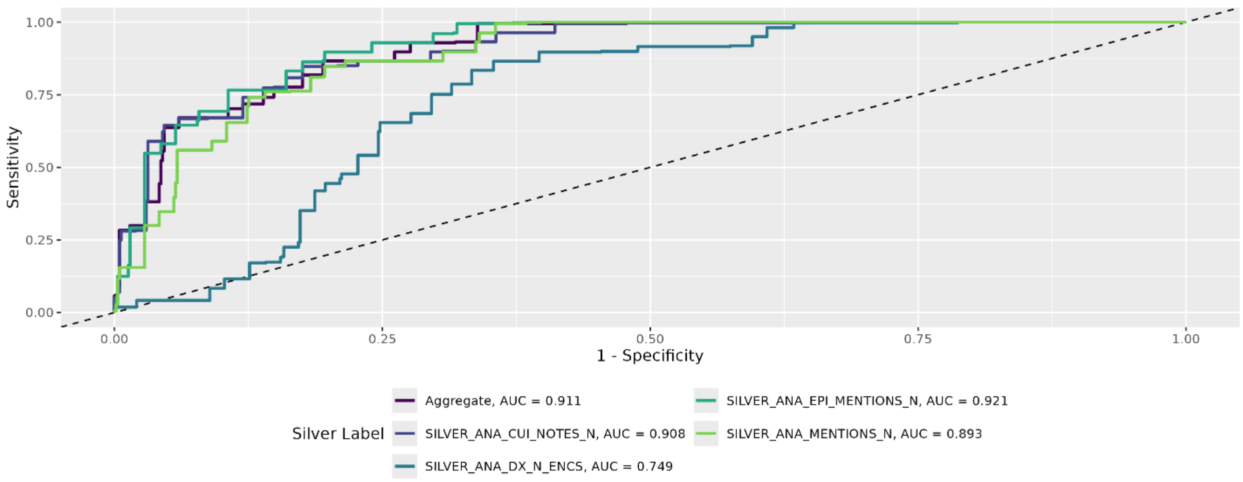

Figure S3: Receiver operating characteristic curves and AUCs for all models trained at VUMC and evaluated on data from KPWA. The silver labels are counts of anaphylaxis diagnosis codes (ANA_DX_N_ENCS), anaphylaxis mentions (ANA_MENTIONS_N), anaphylaxis concept unique identifiers (ANA_CUI_NOTES_N), and anaphylaxis or epinephrine mentions (ANA_EPI_MENTIONS_N).

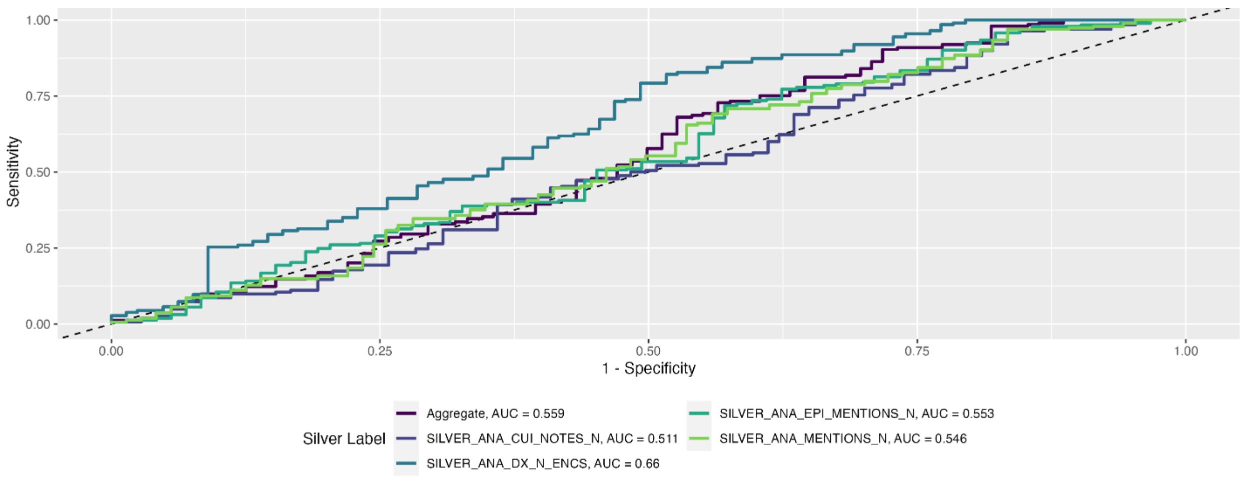

Figure S4: Receiver operating characteristic curves and AUCs for all models trained at KPWA and evaluated on data from VUMC. The silver labels are counts of anaphylaxis diagnosis codes (ANA_DX_N_ENCS), anaphylaxis mentions (ANA_MENTIONS_N), anaphylaxis concept unique identifiers (ANA_CUI_NOTES_N), and anaphylaxis or epinephrine mentions (ANA_EPI_MENTIONS_N).
